# Genetic Interactions of Histone Modification Machinery Set1 and PAF1C with the Recombination Complex Rec114-Mer2-Mei4 in the Formation of Meiotic DNA Double-Strand Breaks

**DOI:** 10.3390/ijms21082679

**Published:** 2020-04-12

**Authors:** Ying Zhang, Takuya Suzuki, Ke Li, Santosh K. Gothwal, Miki Shinohara, Akira Shinohara

**Affiliations:** 1 Institute for Protein Research, Osaka University, Suita, Osaka 565-0871, Japan; 1204zhangying@gmail.com (Y.Z.); likingwsx@gmail.com (K.L.); santoshgothwalbio@gmail.com (S.K.G.); mikis@nara.kindai.ac.jp (M.S.); 2 Graduate School of Science, Osaka University, Suita, Osaka 565-0871, Japan; 3 Department of Advanced Bioscience, Graduate School of Agriculture, Agricultural Technology and Innovation Research Institute, Kindai, University, Nara, Nara 631-8505, Japan; takuya3985@icloud.com; 4 Agricultural Technology and Innovation Research Institute, Kindai, University, Nara, Nara 631-8505, Japan

**Keywords:** meiosis, histone modification, PAF1C, Set1, H3K4 methylation, double-strand break

## Abstract

Homologous recombination is essential for chromosome segregation during meiosis I. Meiotic recombination is initiated by the introduction of double-strand breaks (DSBs) at specific genomic locations called hotspots, which are catalyzed by Spo11 and its partners. DSB hotspots during meiosis are marked with Set1-mediated histone H3K4 methylation. The Spo11 partner complex, Rec114-Mer2-Mei4, essential for the DSB formation, localizes to the chromosome axes. For efficient DSB formation, a hotspot with histone H3K4 methylation on the chromatin loops is tethered to the chromosome axis through the H3K4 methylation reader protein, Spp1, on the axes, which interacts with Mer2. In this study, we found genetic interaction of mutants in a histone modification protein complex called PAF1C with the *REC114* and *MER2* in the DSB formation in budding yeast *Saccharomyces cerevisiae*. Namely, the *paf1c* mutations *rtf1* and *cdc73* showed synthetic defects in meiotic DSB formation only when combined with a wild-type-like tagged allele of either the *REC114* or *MER2*. The synthetic defect of the tagged *REC114* allele in the DSB formation was seen also with the *set1*, but not with *spp1* deletion. These results suggest a novel role of histone modification machinery in DSB formation during meiosis, which is independent of Spp1-mediated loop-axis tethering.

## 1. Introduction

Meiosis is a programed cell division essential for gamete production. A DNA exchange reaction, homologous recombination, is essential for chromosome segregation during meiosis as well as generation of a new combination of parental alleles in the gamete. For the accurate segregation of homologous chromosomes during meiosis I, a reciprocal exchange of two homologous parental DNAs, the crossover, provides a physical linkage between the chromosomes, together with sister chromatid cohesion [1].

Meiotic recombination is highly regulated at different stages during meiosis. The recombination is initiated by the programmed formation of DNA double-strand breaks (DSBs) at specific genomic regions called hotspots [2,3]. In the budding yeast, *Saccharomyces cerevisiae*, recombination hotspots are located in intergenic regions with nucleosome-free features, particularly the 5’-end of genes such as gene promoters, in the genome [4,5]. Moreover, the hotspots are marked with a specific epigenetic modification of histone H3K4 tri-methylation [6,7,8,9]. The H3K4 methylation (H3K4me) is directly catalyzed by a protein complex called COMPASS or Set1C, whose catalytic subunit is Set1 [10]. Set1-mediated H3K4 methylation depends on the other epigenetic marker, histone H2B K123 ubiquitination, which is catalyzed by two ubiquitination enzymes, Rad6 (E2) and Bre1 (E3), with the aid of RNApolymerase II-associated factor I complex (PAF1C) [11]. Indeed, mutations in the *SET1*, *RAD6*, *BRE1*, and *RTF1* (PAF1C component) showed marked reduction of meiotic DSB formation in the genome [7,12,13,14]. Interestingly, these mutants still form significant levels of DSBs, which account for the high spore viability. These residual DSBs in the absence of H3K4 methylation seem to be regulated by a different pathway.

**Figure 1:**
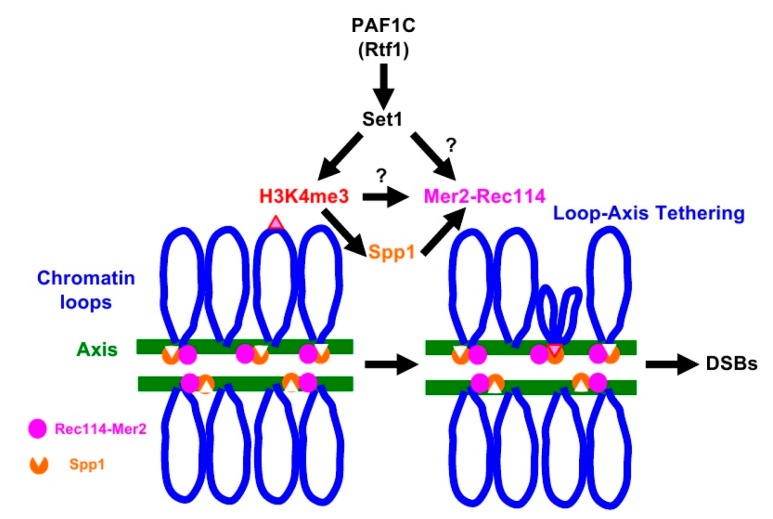
Loop-tethering to the chromosome axis promotes double-strand break (DSB) formation. See details in the text.

A meiosis-specific topoisomerase VI (TopVI) A subunit-like protein, Spo11, directly catalyzes the formation of meiotic DSBs [15]. Importantly, Spo11 needs partner proteins essential to catalyze DSB formation. Similar to TopVI, Spo11 forms a complex with Rec102-Rec104 (a recently identified TopVI B subunit) [16,17] as well as Ski8 [18]. In addition, Mre11-Rad50-Xrs2 (MRX) and Rec114-Mer2-Mei4 (RMM) complexes are also critical for Spo11’s activity [2,3]. The RMM complex binds to distinct regions of chromosomes [19,20], mainly chromosome axes [21], which are spatially unlinked to the recombination hotspots located on chromatin loops [21,22]. N. Kleckner and her colleagues proposed a model for meiotic DSB formation called loop-axis tethering [22]. In the model (See Figure 1, for example), recombination hotspots on the loop interact with chromosome axes enriched for DSB machinery proteins [21]. Indeed, Spp1 protein has been identified as a molecule that mediates the association of the loops with the axis [6,8]. Spp1, although it is a component of COMPASS, binds to the meiotic chromosome axes independently of COMPASS [23,24], and tethers H3K4me-enriched loop regions to the axis through its PHD finger, a conserved binding domain for H3K4 methylation [6,8]. Importantly, Spp1 binds to Mer2 for DSB formation on the axis [6,8].

Although the Spp1 seems to be a key regulator for meiotic DSB formation by Spo11, the *spp1* deletion shows significantly higher levels of DSBs compared to histone modification-defective mutants [6,8,13]. This may imply an additional role of the histone H3K4me for meiotic DSB formation. In this study, we describe unique synthetic interactions between mutations in the histone modification machinery genes and a wild-type-like tagged-allele of *RMM* genes in DSB formation during meiosis. Yeast cells with the deletion of the *RTF1* gene, only when combined with either a *myc*-tagged *REC114* or *MER2* alleles, produce few meiotic DSBs with a big reduction in spore viability. This synthetic defect in DSB formation was also observed in a combination of the tagged *REC114* or *MER2* with the *CDC73* and *SET1* deletion. On the other hand, the *spp1* deletion allele did not show the synthetic defect. These results suggest an Spp1-independent role of PAF1C and Set1 for meiotic DSB formation. We propose that, in addition to its role in loop-axis tethering, histone modification machinery might control the activity of the RMM complex for meiotic DSB formation.

## 2. Results

### 2.1. The rtf1 Shows Synthetic Defects in DSB Formation with REC114-myc

In studies to reveal the relationship of the PAF1C component Rtf1 with DSB formation machinery, we found a strong genetic interaction of the *rtf1∆* mutant with a tagged allele of two components of RMM: *REC114* and *MER2*. As reported [21], *REC114-9myc* (hereafter *REC114-myc*) cells showed similar spore viability to the wild type (Table 1). The *rtf1∆* decreased the viability to 75.3%, consistent with the previous results [13]. When *rtf1∆* was combined with *REC114-myc*, the viability was synergistically reduced to 7.83%, indicating a strong synthetic defect between the *rtf1∆* and *REC114-myc* alleles.

**Figure 2:**
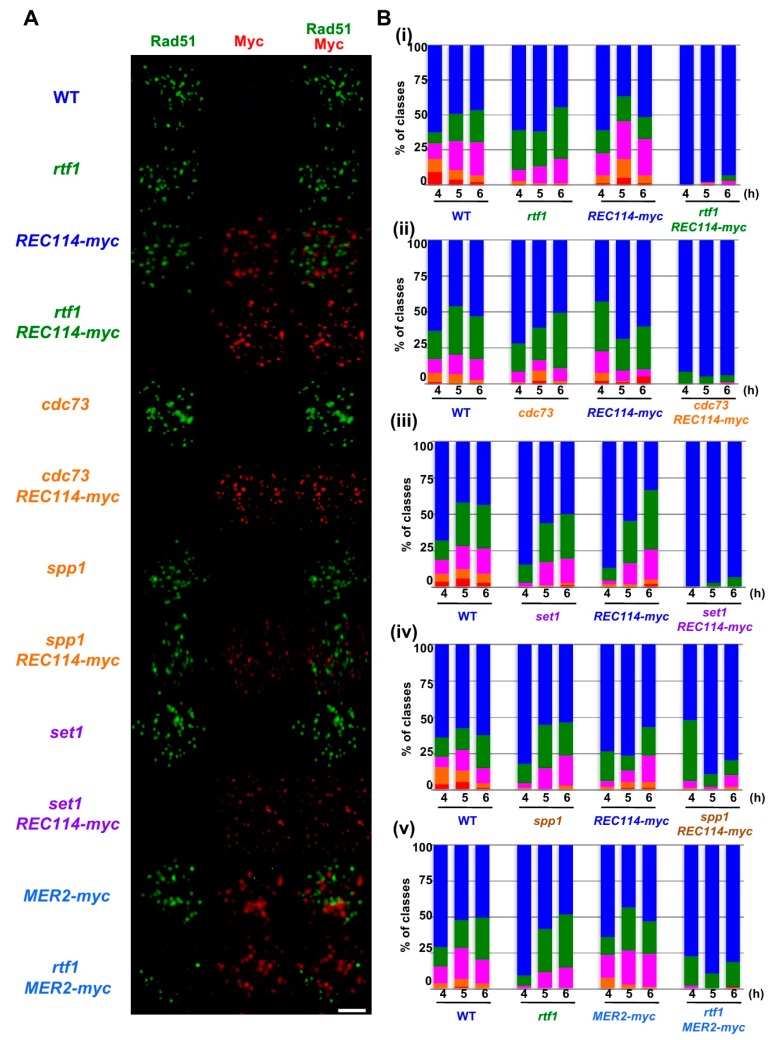
The *REC114-myc* showed a synthetic defect in Rad51-foci formation with histone modification mutations. (**A**) Nuclear spreads from cells undergoing meiosis in various strains were stained with anti-Rad51 (green), anti-myc (red, Rec114/Mer2), and DAPI (blue). The representative images of Rad51/myc/DAPI-staining at 4 h in each strain are shown. Wild type, MSY831/833; *rtf1*, ZYY839; *REC114-myc*, ZYY411; *rtf1 REC114-myc*, ZYY391; *cdc73*, ZYY811; *cdc73 REC114-myc*, ZYY736; *spp1*, ZYY892; *spp1 REC114-myc*, ZYY739; *set1*, ZYY733; *set1 REC114-myc*, ZYY1812; *MER2-myc*, ZYY893; *rtf1 MER2-myc*, ZYY874. Bar = 2 μm. (**B**) The number of Rad51 foci per spread at 4, 5, and 6 h was counted and classified into a spread with less than 5 (Rad51-negative, blue), 6–20 (green), 21–35 (purple), 36–50 (orange), and >50 (red) foci. The graphs show percentages of each class of Rad51-foci numbers. The number is a sum from three independent time courses (*n* = 126; 42 × 3). Data of wild-type, *rtf1,* and *REC114-myc* cells in (i)–(v) were obtained by independent time courses. Statistics for comparison between strains are shown in Table S2.

**Figure 3:**
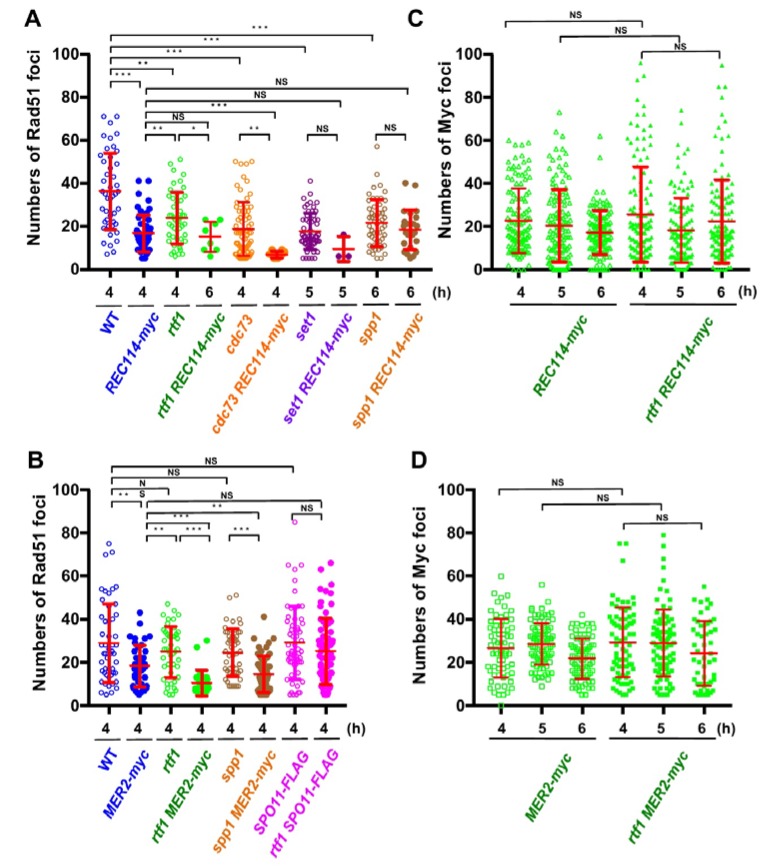
The *REC114-myc* showed a synthetic defect in Rad51-foci formation with histone modification mutations. (**A**,**B**) Distribution of Rad51 foci numbers. The number of Rad51 foci in foci-positive nuclei (with more than 5 foci) in strains with the *REC114-myc* (**A**) and the *MER2-myc* and *SPO11-FLAG* (**B**) at indicated times were counted. At each time point, 42 spreads from three independent time courses (*n* = 126; 42 × 3) were analyzed. * *p* < 0.05; ** *p* < 0.01; *** *p* < 0.001. NS shows Not Significant. The *p* values and statistics for Rad51-foci numbers in foci-positive nuclei are shown in Table S2. The strains used are shown in Figure 1A. *spp1 MER2-myc*, ZYY1030; *SPO11-FLAG* ZYY1031; *rtf1 SPO11-FLAG*, ZYY1032. (**C**,**D**) Distributions of Rec114-myc- and Mer2-myc-foci numbers at 4, 5, and 6 h are shown. In *REC114-myc* (**C**), the number of myc foci in all spreads is shown. In *MER2-myc* (D), the number of myc foci in foci-positive nuclei (with more than 5 foci) is shown. At each time point, 42 spreads from three independent time courses (*n* = 126; 42 × 3) were analyzed. The *p* values and statistics are shown in Table S2.

To know the cause of the reduced spore viability of *rtf1∆ REC114-myc* strain, we analyzed the formation and the repair of meiotic DNA double-strand breaks (DSBs) indirectly by analyzing meiotic Rad51-foci formation. Rad51, a RecA homolog, binds to single-stranded DNAs as a filament [25,26], which can be detected on chromosome spreads of yeast meiotic cells by indirect immuno-staining using an anti-Rad51 antibody [27]. Rad51 punctate staining, called Rad51 foci, is a good marker for DSB formation and repair (Figure 2A). In wild-type cells, Rad51 foci appeared from 3 h incubation with sporulation medium (SPM) and peaked in number at 5 h, and then gradually decreased. After counting the number of Rad51 foci per spread, we classified the numbers at 4, 5, and 6 h incubation in SPM (Figure 2B) and analyzed the distribution of the number in Rad51 foci-positive nuclei; defined by a spread with more than 5 Rad51 foci (Figure 3A,B).

In wild type, at 4 h, the Rad51 foci number was 36.2 ± 3.8 (mean ± S.D.; Figure 3A) per Rad51-foci positive nuclei [27,28]. As reported [13], the *rtf1∆* mutant reduced the Rad51 foci number to 23.1 ± 3.8 at 4 h (*p* value < 0.0001 against wild type, Mann-Whitney U test; Table S2). Although the *REC114-myc* cells showed normal spore viability (Table 1), *REC114-myc* nuclei also showed a decreased number of the foci of 16.7 ± 4.5 at 4 h (*p* value < 0.0001 against wild type, Mann-Whitney U test). The number of Rad51 did not change at later times in the strain (16.2 ± 2.2 and 16.1 ± 8.1 at 5 and 6 h, respectively). These results suggest that the addition of the myc-tag to the C-terminus compromises the function of the Rec114 protein in DSB formation. The *rtf1∆* mutant with *REC114-myc* greatly decreased Rad51 foci formation at 4, 5, and 6 h (Figure 2A). The *rtf1 REC114-myc* showed less than 5% Rad51 foci-positive nuclei while the *rtf1* and *REC114-myc* cells showed more than 35% positive nuclei for Rad51 foci (Figure 2B). Although most of the *rtf1 REC114-myc* cells were negative for Rad51 foci (less than 5 foci; Figure 2B), there were a few nuclei positive for Rad51. At 6 h, the number was 15.1 ± 6.9 (*n* = 6 among 126 nuclei) even in the positive cells (Figure 3A). This indicates that Rtf1 is required for Rad51-foci formation, thus DSB formation, in the presence of the myc-tag on the C-terminus of Rec114. In other words, the C-terminal tagging with myc affects the function of Rec114 in DSB formation when the *RTF1* is absent. Previously, a synthetic defect of the *REC114-myc* in Rad51-foci formation was reported with a deletion mutation of the *RED1* and *HOP1* genes [21], which encode a component of the axial element.

Rec114 is localized to chromosomes during meiosis [19,20]. We checked the localization of Rec114-myc protein on meiotic chromosome spreads (Figure 2A). Consistent with previous studies [19,20], Rec114-myc protein showed punctate staining on the chromosomes. Interestingly, the Rec114-myc foci did not colocalize with Rad51 foci (Figure 2A), supporting the idea that DSB sites marked with Rad51 are spatially separated from Rec114-binding sites on the chromosome axis [21]. Myc foci appeared at 3 h during SPM incubation, stayed on chromosomes at 4, 5, and 6 h, and then disappeared (Figure 3C), consistent with the previous reports [21]. The number of Rec114-myc foci was highly variable from spread to spread with an average of 22.8 ± 15.0 (*n* = 126) at 4 h. The *rtf1* deletion did not affect the formation of Rec114-myc foci, its kinetics, or number (25.6 ± 22.2 at 4 h: *p* = 0.96; Figure 3C and Table S2). Moreover, Rec114-myc did not affect Rtf1-dependent histone H3K4 mono-, di-, and tri-methylation, and H3K79 tri-methylation during meiosis or mitosis (Figure S1A).

### 2.2. The rtf1 Shows a Synthetic Interaction with MER2-myc

Previously, not only the *REC114-myc* but also a Myc-tagged version of *MER*2, *MER2-13myc* (hereafter, *MER2-myc*), showed a synthetic defect with the *red1* and *hop1* mutations in Rad51-foci formation [21]. We also saw genetic interaction of the *rtf1* deletion with the *MER*2*-myc*. As with spore viability, *MER2-myc* slightly decreased to 80.1% (versus wild type, *p* < 0.001, chi-square; Table 1). Importantly, the combination of the *rtf1∆* with *MER2-myc* largely reduced the formation of viable spores (0.5%; Table 1). We analyzed Rad51-foci staining in cells with Mer2-myc (Figure 2A,B). The *MER2-myc* decreased Rad51 focus number to 18.2 ± 9.5 at 4 h relative to the number of wild type cells with 28.9 ± 18.1 (Figure 3B). A similar reduction of Rad51-foci numbers was seen at 5 and 6 h (16.7 ± 8.7 and 12.9 ± 5.6, respectively; Figure 3B). These results suggest that the addition of the myc-tag on the C-terminus may reduce the function of Mer2. Similar to the *rtf1∆ REC114-myc* mutant, *rtf1∆ MER2-myc* showed a large reduction of Rad51-foci positive nuclei (Figure 2B and Figure 3B).

The number of Mer2-myc foci was highly variable among spreads with an average of 28.5 ± 9.5 (among myc-positive nuclei) at 5 h (Figure 3D). The *rtf1* deletion did not affect the formation of Mer2-myc foci, its kinetics, or number (29.3 ± 16.2 at 4 h: *p* = 0.78; Table S2). Moreover, Mer2-myc did not affect Rtf1-dependent histone H3K4 and H3K79 methylations in meiotic and mitotic cells (Figure S1B).

We also checked the interaction of the other component of PAF1C, the *CDC73*, with *REC114-myc* (Figure 2). Similar to the *rtf1* mutant, the *cdc73∆* mutant reduced the steady state number of Rad51 foci to 18.7 ± 12.4 at 4 h (Figure 3A). The *cdc73∆ REC114-myc* diploid cells showed a marked reduction in Rad51 foci-positive cell numbers (Figure 2A,B) and an average number of Rad51 foci (6.8 ± 1.5) lower than either *cdc73∆* or *REC114-myc* strains (Figure 3A), strengthening the idea that a PAF1C-depedent process links RMM function with DSB formation. The *CDC73* deletion did not affect the chromosomal localization of Rec114-myc protein (Figure 2A). Interestingly, the *cdc73∆ REC114-myc* maintained a high spore viability of 61.4% (compared with that of the *rtf1∆ REC114-myc* strain; Table 1).

### 2.3. The set1 Deletion Also Shows a Synthetic Defect with REC114-myc

Rtf1 is critical for Set1(COMPASS)-dependent H3K4 methylation [29]. We combined the *REC114-myc* with the deletion of the *SET1* gene, which encodes an essential catalytic component of the H3K4 methyl-transferase. As reported [12], the steady-state number of Rad51 foci in the *set1∆* mutant was decreased to 17.5 ± 8.5 at 5 h (Figure 2A and Figure 3A). The *set1∆ REC114-myc* cells showed marked reduction in Rad51-foci positive cells during meiosis (Figure 2B) as well as the Rad51 foci number (9.3 ± 5.8 at 5 h; Figure 3A). The *set1* mutation did not affect Rec114-myc localization on the chromosomes (Figure 2A and Figure S3A). These results indicate that the *REC114-myc* shows synthetic defects with the *set1* deletion as well as the *rtf1* deletion in DSB formation. This suggests the role of Set1- and PAF1C-dependent H3K4 methylation in the genetic interaction with RMM.

### 2.4. The spp1 Deletion Does Not Show Genetic Interaction with REC114-myc

A key regulatory structural basis of meiotic chromosomes for DSB formation is the loop-tethering to axis (Figure 1), in which a DSB site marked with histone H3K4 tri-methylation on chromatin loops is tethered to the chromosome axes by a bridging chromosome (axis)-bound Spp1 protein to the H3K4 methylation through the PHD finger of Spp1 [6,8]. Importantly, Spp1 is known to bind to an RMM component, Mer2 [6,8]. Given that the Rtf1 is essential for H3K4 methylation (Figure S1A), similar to the *set1* mutant, the *rtf1* mutant is also defective in loop-axis tethering. To check the role of the Rtf1/Set1–RMM interaction in the loop tethering, we also constructed the *spp1∆ REC114-myc* as well as *spp1∆ MER2-myc* strains ( Figure 2; Figure 3). The *spp1∆* mutant reduced a steady state number of Rad51 foci to 21.4 ± 10.9 at 6 h (Figure 3A), which is similar to those in the *set1* mutant. This is consistent with reduced DSB formation in the mutant [6,8]. The *spp1∆ REC114-myc* cells did not show synthetic defects in Rad51 foci formation (Figure 2A and Figure 3A). The strain decreased slightly the Rad51-foci positive cells and Rad51 foci number compared to the *REC114-myc* and *spp1∆* cells, indicating little genetic interaction between *spp1* and *REC114-myc*. The *spp1∆* did not affect Rec114-myc localization (Figure 2A and Figure S3B). The *spp1∆ REC114-myc* cells showed modest reduction in spore viability of 73.7% (Table 1). The *spp1∆ MER2-myc* mutant showed slight reduction in Rad51-foci formation compared to the *spp1∆*, but higher than *rtf1∆ MER2-myc* (Figure 3B). The *spp1∆ MER2-myc* reduced spore viability to 19.7%. This suggests that the *spp1* mutation is different from the *rtf1* and *set1* in the synthetic interaction with *REC114-myc* and *MER2-myc*. Importantly, different from the *rtf1* and *set1* mutants, the *spp1∆* mutant was proficient in H3K4 mono- and di-methylations as well as H3K79 tri-methylation, and showed reduced H3K4 tri-methylation (Figure S1).

### 2.5. The rtf1 Does Not Show Genetic Interaction with SPO11-FLAG

To determine the specificity of the genetic interaction between Rtf1 and RMM, we analyzed the combination of the *rtf1∆* with a tagged version of the *SPO11, SPO11-FLAG* [30]. The *SPO11-FLAG* cells showed wild-type spore viability (Table 1) and a steady number of Rad51 foci with 29.1 ± 16.9 at 4 h, which is similar to that in wild type (Figure 3B and Figure S2). The *rtf1∆ SPO11-FLAG* showed the similar number of Rad51 foci (25.2 ± 15.4 at 4 h, *p* = 0.164; Figure 3B) to the *SPO11-FLAG*, indicating that the FLAG-tagging of *SPO11* does not compromise the function of the protein in the absence of *RTF1*.

### 2.6. The rtf1∆ REC114-myc dmc1∆ Forms Little DSBs on the Genome

**Figure 4:**
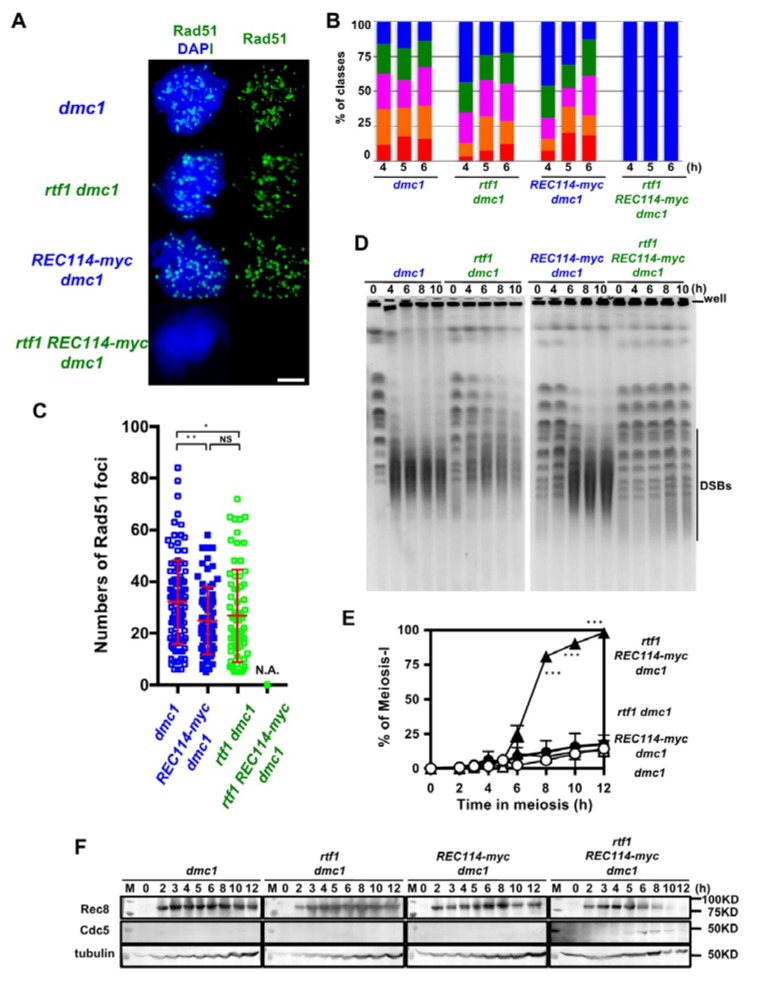
The *rtf1* shows a synthetic defect with *REC114-myc* in DSB formation in a *dmc1* background. (**A**) Nuclear spreads from cells undergoing meiosis from various strains were stained with anti-Rad51 (green) and DAPI (blue). The representative images of Rad51 with DAPI from each strain are shown. *dmc1*, SGY854/855; *rtf1 dmc1*, SGY854/855; *REC114-myc dmc1*, ZYY1016; *rtf1 REC114-myc dmc1*, ZYY1029. Bar = 2 μm. (**B**) The number of Rad51 foci per a spread at 4, 5, and 6 h was counted and categorized into a spread with less than 5 (Rad51-negative, blue), 6–20 (green), 21–35 (purple), 36–50 (orange), and >50 (red) foci. The *dmc1, rtf1 dmc1,* and *REC114-myc dmc1* mutant cells accumulated Rad51 foci during meiosis. On the other hand, the *rtf1 REC114-myc dmc1* mutant showed little Rad51-foci formation. The graphs show percentage of each class of Rad51-foci number. The number is a sum from three independent time courses (*n* = 126; 42 × 3). Statistics for comparison between strains are shown in Table S2. (**C**) Distribution of Rad51 focus number in Rad51-foci positive nuclei (with more than 5 foci) in strains. The *dmc1, rtf1 dmc1,* and *REC114-myc dmc1* mutant cells accumulated Rad51 foci during meiosis. On the other hand, the *rtf1 REC114-myc dmc1* mutant showed little Rad51-foci formation. The *p* values and statistics for Rad51-foci number in foci-positive nuclei are shown in Table S2. * and ** indicate *p* < 0.05 and *p* < 0.01, respectively. NS shows Not Significant. Statistics for comparison between strains are shown in Table S2. (**D**) DSB repair on chromosomes were analyzed by the pulsed field gel electrophoresis (PFGE). Chromosomes were separated by PFGE and stained with EtBr. The same strains were used as in (**A**). The *dmc1* as well as *rtf1 dmc1* and *REC114-myc dmc1* mutant cells showed normal chromosome ladders with intact chromosomes at 0 h (pre-meiosis) and loose bands of intact chromosomes with accumulation of smear bands with less than 500 kb after 4 h. The *rtf1 REC114-myc dmc1* mutant showed almost intact chromosome bands from 4 to 10 h. (**E**) Meiotic cell cycle progression. Entry into meiosis I and II in the wild-type and various mutant cells were analyzed by DAPI staining. The number of DAPI bodies in a cell was counted. Graphs show the percent of cells that completed MI and/or MII at the indicated times. More than 200 cells were counted at each time point. Error bars show S.D. (three independent time courses). The *dmc1, rtf1 dmc1,* and *REC114-myc dmc1* mutant cells showed an arrest during meiosis. On the other hand, the *rtf1 REC114-myc dmc1* mutant carried our meiosis I division. Difference between *rtf1 dmc1* and *rtf1 REC114-myc dmc1* strains were compared. *** indicates *p* < 0.001. Statistics for comparison between strains are also shown in Table S2. (**F**) Cell lysates obtained from various *dmc1* mutant cells at indicated times in meiosis were analyzed by Western blotting using anti-Rec8 (top), and anti-Cdc5 antibodies (middle). Rec8 was induced at 2 h after the induction of meiosis and accumulated in the *dmc1, rtf1 dmc1,* and *REC114-myc dmc1* mutant cells. Cdc5 was not expressed in the *dmc1, rtf1 dmc1,* or *REC114-myc dmc1* cells. On the other hand, in the *rtf1 REC114-myc dmc1* mutant, Rec8 disappeared after 10 h and Cdc5 was expressed from 6 h. Tubulin was used as a control (bottom). “M” shows a marker.

The above studies were done in a wild-type background, in which DSBs are turned over. To exclude the possibility that the synthetic defect between *rtf1* and *REC114-myc* in DSB formation is due to abnormal DSB turnover, such as rapid repair, we introduced the deletion of the *DMC1* gene, which encodes a Rad51 homolog essential for meiotic DSB repair [27]. The *dmc1∆* single mutant accumulated Rad51 foci (Figure 4A,B) with an average number of 32.3 ± 16.3 per a foci-positive spread at 4 h during meiosis. The *REC114-myc dmc1∆* and *rtf1∆ dmc1∆* accumulated Rad51 foci with a slightly reduced number relative to the *dmc1∆* (25.0 ± 12.3, and 26.9 ± 18.1, respectively; *p* value < 0.05 in both cases against wild type, Mann-Whitney U test) at 4 h (Figure 4C). The *rtf1∆ REC114-myc dmc1∆* formed few Rad51 foci at any time points (Figure 4A–C).

We confirmed DSB formation by the physical analysis of DSBs at a recombination hotspot, the *HIS4-LEU2* locus. The *dmc1∆* mutant accumulated DSBs at the locus as reported [27]. As shown previously [13], the *rtf1∆ dmc1∆* mutant decreased by ~1/3 the DSB levels relative to the *dmc1∆* mutant (Figure S4). The *REC114-myc* also showed similar reduction of DSBs to the *rtf1∆ dmc1∆* mutant. Unexpectedly, the *rtf1∆ REC114-myc dmc1∆* mutant also formed reduced levels of DSBs similar to those in *rtf1∆ dmc1∆* and *REC114-myc dmc1∆* mutants (Figure S4).

To solve the discrepancy in results between Rad51-foci counts and DSB frequencies at the *HIS4-LEU2* locus, we analyzed fragmented chromosomes induced by meiotic DSBs by pulsed-field gel electrophoresis (PFGE). In the *dmc1∆* mutant, after 4 h, most of the full-length chromosomes were fragmented into small fragments with a size of ~50–500 kbp (Figure 4D). On the other hand, the *rtf1∆ dmc1∆* mutant showed reduced frequencies of fragments relative to the *dmc1*. The *REC114-myc* showed slight reduced levels of the fragments compared to the *dmc1∆*. Importantly, in the *rtf1∆ REC114-myc dmc1∆* mutant, all chromosomes were nearly intact from 4 to 10 h. This indicated little DSB formation along chromosomes when the *rtf1∆* was combined with the *REC114-myc* allele, confirming the synthetic defect in DSB formation as shown by Rad51 staining.

### 2.7. The rtf1 and REC114-myc Alleviates dmc1 Arrest

As expected, the combination of the *rtf1∆* and *REC114-myc*, but neither *rtf1∆* nor *REC114-myc* alleles alone, alleviated the prophase-I arrest induced by the *dmc1* deletion (Figure 4E), supporting fewer DSBs introduced in the *rtf1∆ REC114-myc dmc1∆* mutant. This was confirmed by the expression of Cdc5 polo-like kinase, which is expressed after the pachytene exit, in the *rtf1∆ REC114-myc dmc1∆* mutant (Figure 4F) similar to the cells with wild-type *DMC1* (Figure S5), while there was little expression of Cdc5 in the *dmc1∆* mutant due to the mid-pachytene arrest [31]. Moreover, Rec8 levels were persistent in the *dmc1∆*, *rtf1∆ dmc1∆,* and *dmc1∆ REC114-myc dmc1∆* mutants while the full length of Rec8 was decreased after 8 h in the *rtf1∆ REC114-myc dmc1∆* mutant, due to the cleavage of Rec8 in the onset of anaphase I (Figure 4F and Figure S5).

## 3. Discussion

In this paper, we describe unusual genetic interaction among alleles of genes involved in DSB formation. Particularly, some combination of the deletion of genes involved in histone modification for DSB formation with two tagged alleles of genes working together with Spo11 showed synergistic defects in meiotic DSB formation. When combined with a C-terminus myc-tagged allele of either *REC114* or *MER2*, the deletion of a *PAF1C* gene, the *RTF1*, whose single mutation moderately decreases DSB frequencies, resulted in fewer meiotic DSBs. This shows that Rtf1 is essential for DSB formation when either Rec114 or Mer2 bears a C-terminus myc-tag. In other words, in the absence of the Rtf1, the C-terminal tag of either Rec114 or Mer2 is harmful for DSB formation.

We also observed a similar synthetic defect between the deletion of the other components of PAF1C, *CDC73*, and the *REC114*-myc. These results suggest the role of PAF1C in the interaction with RMM. On the other hand, the *rtf1* deletion did not show any genetic interaction with a tagged allele of *SPO11*. These results suggest a specific connection of PAF1C with RMM functions. We also showed a synthetic defect between the *set1* deletion and *REC114-myc*. Set1, a component of histone H3K4 methy-transferase, works downstream of PAF1C through histone H2BK123 ubiquitination (Figure 1) [10]. Given that the *rtf1*, *cdc73*, and *set1* mutants are defective at H3K4 tri-methylation (Figure S1), our results suggest the important role of H3K4 methylation in the synthetic defect between histone modification machinery and RMM.

H3K4 methylation plays a critical role in meiotic DSB formation and the role is evolutionarily conserved from yeasts to human [32]. H3K4 methylation on recombination hotspots on chromatin loops is tethered to the chromosome axes through capturing of the methylation by axis-bound protein Spp1, which interacts with Mer2, which is critical for DSB formation (Figure 1) [6,8]. The synthetic defects we observed between the H3K4 modification machinery and RMM complex suggest the role of RMM in DSB formation in the absence of loop tethering to the axis. We checked the genetic interaction between *SPP1* deletion, which disrupted the tethering with H3K4 methylation, and *REC114-myc*, but we could not see synthetic defects in DSB formation. Although Spp1 is a component of the H3K4 methyl-transferase complex, the *spp1* deletion mutant retains moderate levels of H3K4 methylation [6,8] (Figure S1). This suggests that the absence of loop-tethering to the axis does not affect DSB activity of the *REC114-myc* cells. One likely possibility is that, in the addition to the role in loop tethering, H3K4 methylation plays a role in DSB formation through the control of activity of RMM (Figure 1). Alternatively, Set1-complex together with PAF1C may catalyze the methylation of proteins involved in DSB formation, such as RMM. It was shown that Set1, together with PAF1C, Bre1, and Rad6, methylates a kinetochore protein, Dam1 [33,34].

Although the *rtf1* and *set1* showed similar synthetic defects in DSB formation with the *REC114-myc*, the two mutations were different in spore viability. The *set1 REC114-myc* mutant showed high spore viability of 76% while the *rtf1 REC114-myc* mutant reduced the viability to 7.8%. Similar to the *rtf1 REC114-myc* mutant, the *set1 REC114-myc* mutant formed fewer DSBs. This suggests the presence of a novel mechanism to support proper chromosome segregation during meiosis to ensure high spore viability in the *set1 REC114-myc* but not in the *rtf1 REC114-myc* mutant.

Our results described here are consistent with a previous study that showed that the *REC114-myc* and *MER2-myc* alleles also have a synthetic interaction with deletion mutations of the *RED1* and *HOP1* genes [21], which encode a component of the axial element. In future, it will be interesting to check the genetic interaction of mutants between histone modification machinery and axis components.

**Figure 5:**
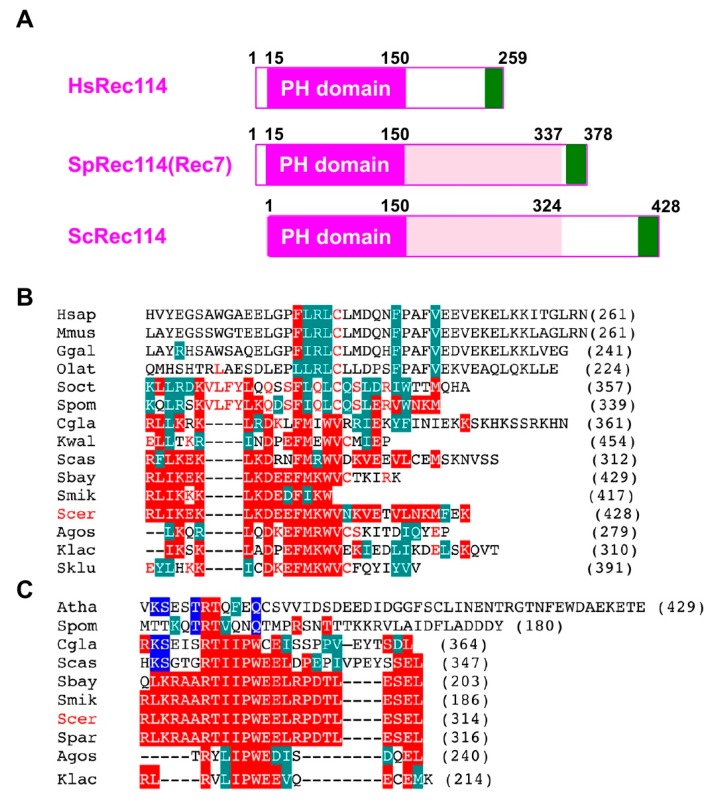
Sequence conservation of the C-terminal regions of Rec114 and Mer2. (**A**) Schematic comparison of human Rec114, fission yeast Rec114 (Rec7), and budding yeast Rec114. The PH domain is shown in a purple box. A newly identified conserved region of the C-terminus is shown in a green box. (**B**) Sequence alignment of a C-terminal region of the Rec114 orthologs from various species: *H. sapiens*, *M. musculus*, *G. gallus*, *O. latipes* (medaka), *S. octosporus*, *S. pombe*, *C. glabrata*, *K. waltii*, *S. castellii*, *S. boyanus*, *S. mikatae*, *S. cerevisiae*, *S. paradoxus*, *A. gossypii*, *K. lactis*, *S. kluyveri*. Alignment was produced by MUSCLE (http://www.drive5.com/muscle/) and is shown in CLUSTALW format. Identical and homologous amino acids with ScRec114 are shown in red and green boxes, respectively. Identical amino acids with SpRec114 (Rec7) are shown in red letters. (**C**) Sequence alignment of a C-terminal region of the Mer2 ortholog from various species: *A. thaliana*, *S. pombe*, *C. glabrata*, *S. castellii*, *S. boyanus*, *S. mikatae*, *S. cerevisiae*, *S. paradoxus*, *A. gossypii*, *K. lactis*. Identical and homologous amino acids with ScMer2 are shown in red and green boxes, respectively. Alignment was produced by MUSCLE program and is shown in CLUSTALW format as shown in (**B**). Identical amino acids with *Arabidopsis* Mer2 (PRD3) are shown in blue boxes.

The fact that the C-terminal myc-tag on Rec114 and Mer2 affects function, suggests an important role of the C-terminal region of proteins. Recent studies identified orthologs of *S. cerevisiae* Rec114 and Mer2 proteins from different yeasts to mammals [35,36]. The N-terminal ~140 aa of Rec114 contains a conserved PH domain (Figure 5A), whose crystal structure adapts β-sandwich folds [35,36]. We found that an ~33 aa region in the Rec114 C-terminus is also conserved among some yeasts (Figure 5B). This region also shares similarity to the C-terminal region of Rec114 in fission yeast (a.k.a. Rec7) and vertebrate Rec114 orthologs (Figure 5B). Moreover, the C-terminus of *S. cerevisiae* Mer2 protein is conserved among some yeast species (Figure 5C) with weak homology to Mer2 orthologs in *S. pombe* (Rec15) and in *Arabidopsis thaliana* (PRD3). These regions of both Rec114 and Mer2 are rich in acidic and basic amino acids. These support an idea that the C-terminal regions of Rec114 and Mer2 are very important for its activity, such as in protein–protein interactions. Interestingly, Kumar et al., (2010) showed the C-terminal region of mouse Rec114 binds to another RMM component, Mei4 [37]. The C-terminal myc-tagging of Rec114 and Mer2 might affect such a function.

## 4. Materials and Methods

### 4.1. Strains and Strain Construction

All strains described here are derivatives of SK1 diploid strains, MSY831/833 (MATα/MAT**a**, ho::LYS2/”, lys2/”, ura3/”, leu2::hisG/”, trp1::hisG/”). For the dmc1 mutant, NKY1551 (MATα/MAT**a**, ho::LYS2/”, lys2/”, ura3/”, leu2::hisG/”, his4X-LEU2-URA3/”, his4B-LEU2/”, arg4-bgl/arg4-nsp) background was used. REC114-9myc and MER2-13myc were a gift from Dr. F. Klein. SPO11-3FLAG was provided by Dr. K. Ohta. Strain genotypes are given in Table S1.

### 4.2. Antisera and Antibodies

Rabbit anti-Rad51 (1:200) [26] and mouse anti-myc (Nakarai, Kyoto, Japan, MC045; 1:200) antibodies for cytology have been described previously [28,38]. Secondary antibodies conjugated with Alxea488 and Alexa594 dyes (Themo Fishers, Waltham, MA, USA) were used for the detection of the primary antibodies. For Western blotting, the following antibodies were used: anti-Rec8 (1:200) [39]; anti-Cdc5 (Santra Cruz, Dallas, TX, USA, sc-33635, 1:1000), 1:200; anti-histone H3K4 mono-methyl (Abcam, Cambrdige, UK, ab8895; 1:1000), di-methyl (Millipore, Burlington, MA, USA, 07-030; 1:1000), tri-methyl (Abcam, ab8580; 1:1000); anti-histone H3K79 tri-methyl (Abcam, ab2621; 1:1000).

### 4.3. ImmunoStaining

Immunostaining of chromosome spreads was performed as described previously [28,37]. Stained samples were observed using an epi-fluorescence microscope (BX51; Olympus, Tokyo, Japan) with a 100× objective (NA1.3). Images were captured by CCD camera (CoolSNAP; Roper, Sarasota, FL, USA) and afterwards processed using IP lab and/or iVision (Sillicon, Austin, TX, USA) and Photoshop (Adobe, San Jose, CA, USA) software tools.

### 4.4. Southern Blotting

Southern blotting analysis was performed with the same procedure as previously described [40]. Genomic DNAs were digested with *Pst*I (for meiotic DSB, lower panels). The probe for Southern blotting was “Probe 291” for DSB detection as previously described [40]. The image was captured by using Typhoon FLA7000 (GE healthcare, Chicago, IL, USA). Image Quant software (GE healthcare) was used for quantification of the bands of DSB-I.

### 4.5. Western Blotting

Western blotting was performed for cell lysates extracted by the TCA method. After being harvested and washed twice with 20% TCA, cells were roughly disrupted with glass beads by a Yasui Kikai disrupter (Yasui Kikai Co Ltd., Osaka, Japan). Protein precipitation recovered by centrifuge at 3000 rpm for 5 min was suspended in SDS-PAGE sample buffer adjusting to pH 8.8 and then boiled at 95 °C for 2 min.

### 4.6. Pulsed Field Gel Electrophoresis

For pulsed field gel electrophoresis (PFGE), chromosomal DNAs were prepared in agarose plugs as described in [12,13] and run at 14 °C in a CHEF DR-III apparatus (BioRad, Hercules, CA, USA) using the field 6 V/cm at a 120° angle. Switching times followed a ramp from 15.1 to 25.1 s. Gels were stained with EtBr for 30 min and de-stained overnight. The images were captured by LAS4000 (GE healthcare).

### 4.7. Statistics

Means ± Standard Deviation (S.D.) values are shown. Datasets were compared using the Student *t*-test, Mann–Whitney U test, or Wilcoxon matched pairs rank test. χ^2^-test was used for proportion. The *p* values were obtained using Prism 7 (GraphPad) or Excel (Microsoft). Multiple test correction was done with Bonferroni’s correction. *, **, and *** show *p* values of <0.05, <0.01, and <0.001, respectively.

## Figures and Tables

**Table 1:** Spore viability and distribution of viable spores.

Strain Genotype	Distribution of Viable Spores per a Tetrad (*n* = 300)					Spore Viability (%)
	4 Viable	3 Viable	2 Viable	1 Viable	0 Viable	
Wild type	268	28	4	0	0	97.0
*rtf1*	108	116	54	16	6	75.3
*REC114-myc*	274	13	8	0	5	95.9
*rtf1 REC114-myc*	12	4	12	10	263	7.83
*MER2-myc*	223	12	15	3	47	80.1
*rtf1 MER2-myc*	0	0	3	0	297	0.5
*cdc73*	286	13	1	0	41	98.8
*cdc73 REC114-myc*	107	62	52	19	58	61.4
*set1*	248	45	5	2	0	94.9
*set1 REC114-myc*	194	31	21	2	52	76.1
*spp1*	255	36	9	0	0	95.5
*spp1 REC114-myc*	184	35	17	9	54	73.7
*spp1 MER2-myc*	40	11	21	1	227	19.7
*SPO11-FLAG*	286	13	1	0	0	98.8
*rtf1 SPO11-FLAG*	142	96	44	14	4	79.8

## Supplementary Materials

The following are available online at https://www.mdpi.com/1422-0067/21/8/2679/s1, Figure S1: Western blotting analysis of histone modifications in various strains during meiosis, Figure S2: Rad51-staining in *SPO11-Flag* strains, Figure S3: Numbers of Rec114-myc foci in various strains, Figure S4: DSB formation at the *HIS4-LEU2* loci in various *dmc1* strains, Figure S5: Western blotting analysis of meiosis progression in various strains, Table S1: Strain list, Table S2: Statistics Summary.
