# Supplementary material for: Genetic Interactions of Histone Modification Machinery Set1 and PAF1C with the Recombination Complex Rec114-Mer2-Mei4 in the Formation of Meiotic DNA Double-Strand Breaks
Source: Int J Mol Sci. 2020 Apr 12;21(8):2679. doi: 10.3390/ijms21082679 (PMC7215556; doi:10.3390/ijms21082679)
Supplement: Supplementary file 1 [file ijms-21-02679-s001.zip › Supplementary Table S1 and Figure S1-S5.pdf]

Table S1. Strain List

MSY831/833	*MAT**a**/* α, *ho::LYS2/", lys2/", ura3/", leu2::hisG/", trp1::hisG/"*
NKY1551	*MAT**a**/* α, *ho::LYS2/", lys2/", ura3/", leu2::hisG/", his4X-LEU2(BamHI)-URA3/his4B-LEU2(MluI), arg4-bgl/arg4-nsp*
ZYY389	MSY831/833 with *rtf1::TRP1/"*
ZYY391	MSY831/833 with *rtf1::TRP1/", REC114-myc::KamMX6/"*
ZYY411	MSY831/833 with *REC114-myc::KamMX6/"*
ZYY733	MSY831/833 with *set1::URA3/"*
ZYY812	MSY831/833 with *set1::URA3/", REC114-myc::KamMX6/"*
ZYY874	MSY831/833 with *rtf1::TRP1/", MER2-myc::KamMX6/"*
ZYY893	MSY831/833 with *MER2-myc::KamMX6/"*
ZYY811	MSY831/833 with *cdc73::TRP1/"*
ZYY736	MSY831/833 with *cdc73::TRP1/", REC114-myc::KamMX6/"*
ZYY892	MSY831/833 with *spp1::HYGMX6/"*
ZYY1030	MSY831/833 with *spp1::HYGMX6/", MER2-myc::KamMX6/"*
ZYY1031	MSY831/833 with *rtf1::TRP1/"*
ZYY1032	MSY831/833 with *rtf1::TRP1/", SPO11-FLAG::KamMX6*
SGY854/855	NKY1551 with *dmc1::URA3/"*
SGY859/860	NKY1551 with *rtf1::TRP1/", dmc1::URA3/"*
ZYY1016	NKY1551 with *REC114-myc::KamMX6/" dmc1::URA3/"*
ZYY1029	NKY1551 with *REC114-myc::KamMX6/", rtf1::TRP1/", dmc1::URA3/"*

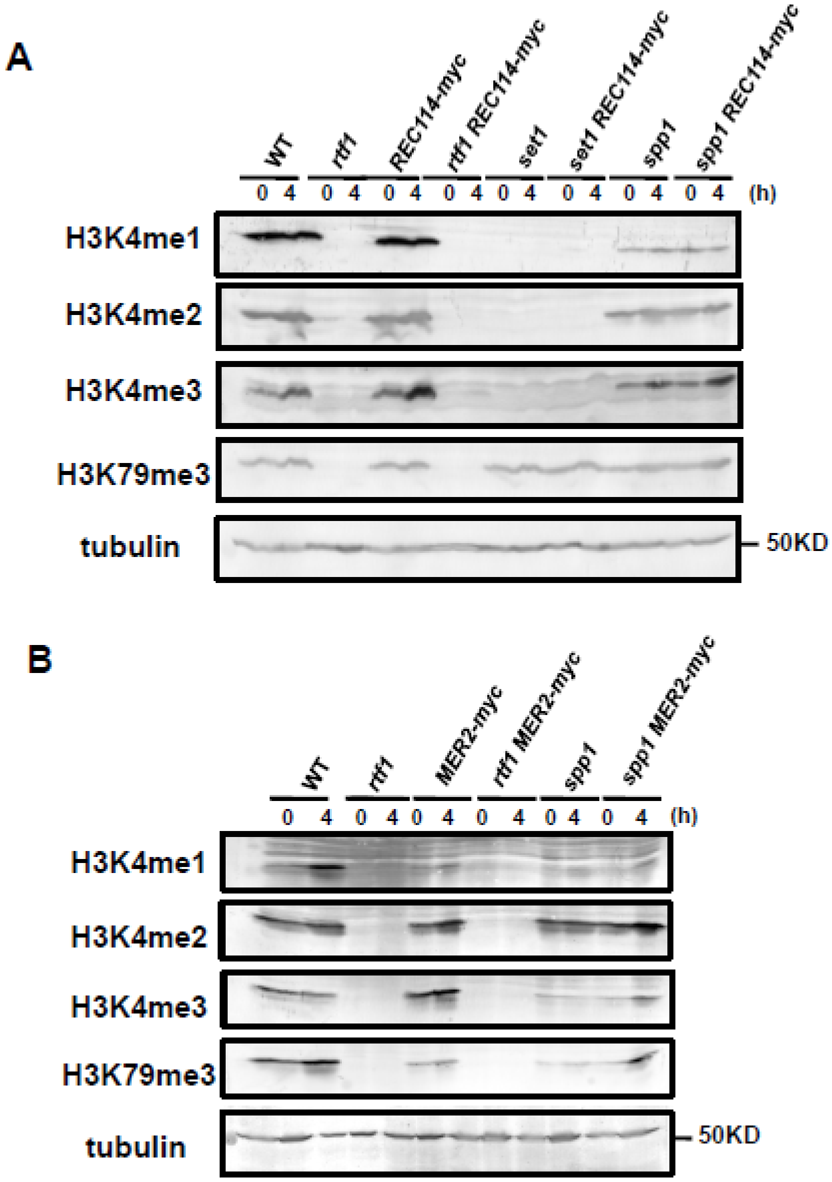

Figure S1. Western blotting analysis of histone modifications in various strains during meiosis**.** Cell lysates obtained from wild type and various mutant cells in meiosis at 0 and 4 h in meiosis were analyzed by Western blotting using anti-histone H3K4 mono-, di- and tri-methylation as well as anti-histone H3K79 tri-methylation. Tubulin was used as a control. The *rtf1* mutant almost abolished H3K4 mono-, di- and tri-methylation and anti-histone H3K79 tri-methylation. The set1 mutant is defective in H3K4 mono-, di- and tri-methylation, but not in H3K79 tri-methylation. The *spp1* mutant shows reduced level of H3K4 methylations. The myc-tag on either the *REC114* (A) or *MER2* (B) did not affect the status of H3K4 and K79 methylations in various mutants. Wild type, MSY831/833; *rtf1*, ZYY839; *REC114-myc*, ZYY411; *rtf1 REC114-myc*, ZYY391; *spp1*, ZYY892; *spp1 REC114-myc*, ZYY739; *set1*, ZYY733; *set1 REC114-myc*, ZYY1812; *MER2-myc*, ZYY893; *rtf1 MER2-myc*, ZYY874; *spp1 MER2-myc*, ZYY1030.

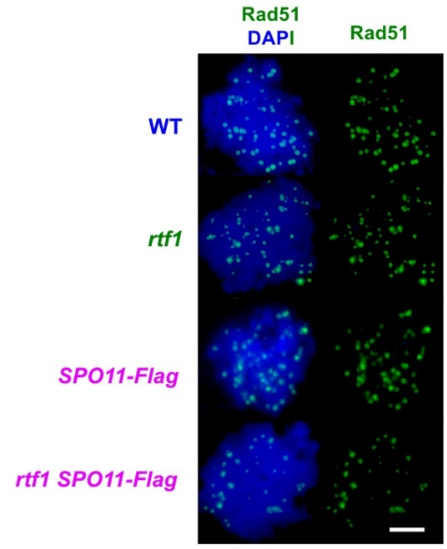

Figure S2. Rad51 staining in the *SPO11-Flag* strains. Nuclear spreads from cells undergoing meiosis at various strains were stained with anti-Rad51 (green) and DAPI (blue). The representative images of Rad51-staining from each strain are shown. Wild type, MSY831/833; *rtf1*, ZYY839; *SPO11-Flag*, ZYY1031; *rtf1 SPO11-Flag*, ZYY1032. Bar = 2 μm.

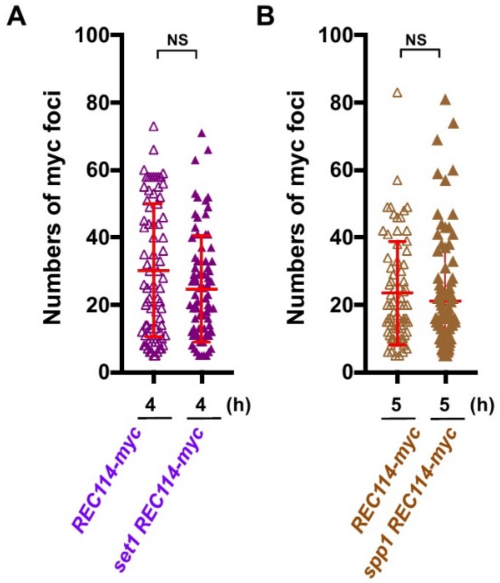

Figure S3. Numbers of Rec114-myc foci in various strains. Distribution of Rec114-myc-focus number is shown. The number of myc foci in focus-positive nuclei (with more than 5 foci) is shown. At each time point, 42 spreads from three independent time courses (n=126; 42X3) were examined. *REC114-myc*, ZYY411; *set1 REC114-myc*, ZYY1812; *spp1 REC114-myc*, ZYY739.

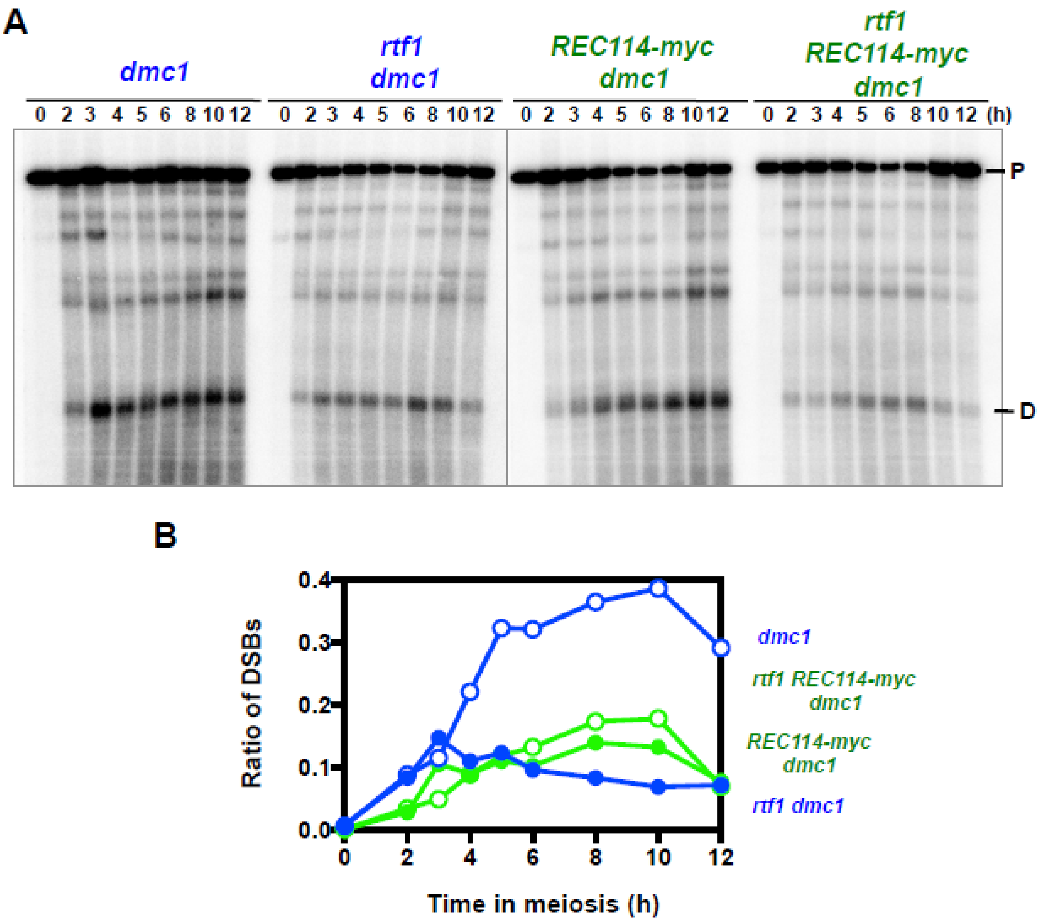

Figure S4. DSB formation at the *HIS4-LEU2* locus in various *dmc1* strains. DSB repair at the *HIS4-LEU2* locus were analyzed by Southern blotting (A). Genomic DNAs were prepared and digested with *Pst*I. On each blot, ratio of DSB bands at site I of the locus ("D", in the right) to parental bands (P) was calculated and plotted (B). *dmc1Δ* (SGY854/855), *rtf1Δ dmc1Δ* (SGY859/860), *REC114-myc dmc1Δ* (ZYY1016) and *rtf1Δ REC114-myc dmc1Δ* (ZYY1029) cells were used. In the *dmc1Δ*, DSB fragments appeared at 2 h and accumulated during further incubation. The *rtf1Δ dmc1Δ*, *REC114-myc dmc1Δ* and *rtf1Δ REC114-myc dmc1Δ* mutants showed ~3-fold reduced DSBs compared to the *dmc1Δ* mutant.

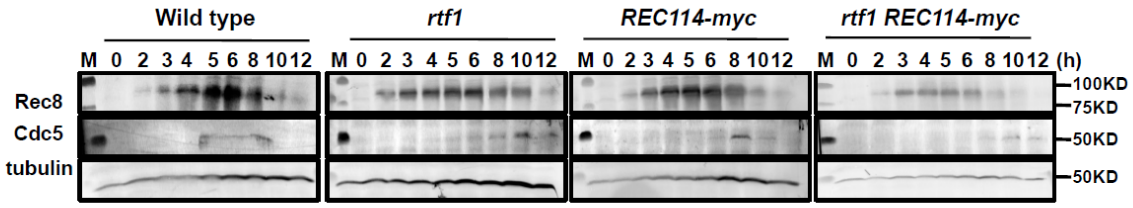

Figure S5. Western blotting analysis of meiosis progression in various strains. Cell lysates obtained from wild type (MSY831/833) and various mutant cells in meiosis at indicated times in meiosis were analyzed by western blotting using anti-Rec8 (top), and anti-Cdc5 antibodies (middle). Tubulin was used as a control (bottom). "M" shows a marker. Wild type, MSHY831/833; *rtf1*, ZYY839; *REC114-myc*, ZYY411; *rtf1 REC114-myc*. Rec8 is induced at 2 h after the induction of meiosis and disappears at late time points. Cdc5 is transiently expressed after pachytene stage during meiosis. The *rtf1*, *REC114-myc*, and *rtf1 REC114-myc* cells delay the expression of Cdc5 compared to wild-type cells.
